# Sudden Death in the Young: A Nationwide Prospective Epidemiological Study of Sudden Death in Young People Aged 1–35 Years in the Mediterranean Island of Cyprus

**DOI:** 10.1111/jce.70037

**Published:** 2025-08-06

**Authors:** Loizos Antoniades, Constantinos Antoniades, Hera Moustra Heracleous, Georgia Daniel, Alexandros Protonotarios, Aris Anastasakis, Adalena Tsatsoppoulou, Petros Agathangelou

**Affiliations:** ^1^ Cyprus Institute of Cardiomyopathies and Inherited Cardiovascular Diseases Nicosia Cyprus; ^2^ Department of Cardiology Nicosia General Hospital Nicosia Cyprus; ^3^ European University Cyprus Nicosia Cyprus; ^4^ Institute of Cardiovascular Science University College London London UK; ^5^ Unit of Inherited and Rare Cardiovascular Diseases Onassis Cardiac Surgery Centre Athens Greece; ^6^ Nikos Protonotarios Medical Centre Naxos Greece

**Keywords:** cardiomyopathies, Cyprus, epidemiology, sudden death, young

## Abstract

**Background and Aims:**

This study aimed to explore the etiology, incidence, and epidemiological characteristics of sudden death (SD) cases among individuals aged 1–35 years, through a systemic evaluation of all SDs in Cyprus over a 11‐year period.

**Methods and Results:**

From 2005 to 2015, all cases of SDs involving individuals aged 1–35 years, who were citizens or permanent residents of the Republic of Cyprus, were recorded and studied. The incidence of SD in young individuals in Cyprus was 2.16 cases per 100 000 people annually. Out of the 74 recorded cases, cardiac causes were identified in 43.24% (*n* = 32) of SD cases, with 28.38% (*n* = 21) attributed to noncardiac factors. Cardiac causes predominated in males (*n* = 25/50, 50.00%), while noncardiac causes were more prevalent in females (*n* = 12/24, 50.00%). Among all SD cases, cardiomyopathies, particularly hypertrophic cardiomyopathy, accounted for 24.32% (*n* = 18) cases, followed by pulmonary embolism (*n* = 8, 10.81%) and viral myocarditis (*n* = 7, 9.46%) as the second and third leading causes, respectively. Other causes of SDs were myocardial infarction (*n* = 5, 6.76%), and hemorrhagic stroke (*n* = 4, 5.41%). Aortic aneurysm rupture, adrenal hemorrhage, and pulmonary aspiration each constituted 4.05% (*n* = 3) of SD cases. The cause of death remained indeterminate in 28.38% (*n* = 21) of SD cases.

**Conclusions:**

SD in the young is predominantly of cardiac origin, although a significant proportion of cases is also attributed to noncardiac causes. Despite thorough post‐mortem examinations, including microscopic pathology, histological, and immune‐histological analyses, a considerable number of SD cases remain unclear.

## Graphical abstract

Epidemiological profile of sudden death in young individuals aged 1–35 years in Cyprus: Cardiac causes account for the majority of cases, with cardiomyopathies being the leading cause of sudden cardiac death. A high proportion of unexplained deaths highlights the need for further targeted genetic evaluation.

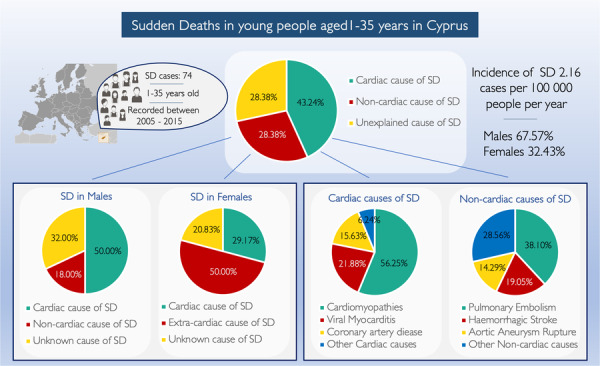

## 1 Introduction

Sudden death (SD) of a young individual is an uncommon and tragic event that has an immense impact not only on the victims' family but also on their friends and the surrounding community because of its unexpected and catastrophic nature. Epidemiological studies of SD in several European countries [1, 2, 3, 4, 5, 6, 7] have delved into the etiological complexity and frequency of SD and have offered important information into the prevalence and etiology of SD in young people. While cardiovascular events, particularly inherited cardiovascular diseases, are commonly identified as the primary cause of SD in young individuals, in a large number of cases, the cause of SD remains unclear.

Existing epidemiological data on SDs in young people, from various countries, predominantly rely on retrospective analyses of post‐mortem data. Epidemiological studies and scientific records have shown that, on average, there are 0.8–8.5 cases of SD per 100 000 young people per year [1, 2, 3, 4, 5, 6, 7]. This large variation in the frequency of SDs can be attributed to the differences in study methodologies but also to local and national variations. The knowledge of clinical and epidemiological characteristics of SDs in a country is a crucial prerequisite to successfully identifying the causative factors and formulating a robust strategy of how to prevent and manage these cases.

There are relatively few studies investigating the frequency and etiology of SD in young people at a national level [4]. Such studies are valuable as they capture all cases within a defined population, enabling comprehensive insights into epidemiological patterns and potential hereditary influences. Situated at the northeastern end of the Mediterranean basin, Cyprus is the third largest island in the region, covering 3572 square miles with a population of 920 701, including 310 287 individuals aged 1–35 years. A legal framework mandating the recording and investigation of all sudden deaths enabled the prospective inclusion of all SD cases in young people across the island. By focusing on Cypriot citizens, this study captures the vast majority of the population, providing data comparable to a regional study while preserving the advantages of a national‐level approach. The selected age group (1–35 years) and analysis of SD etiology were aligned with similar European studies to allow meaningful comparisons.

## 2 Methods

### 2.1 Study Design

In 2004, an Inherited Cardiovascular Diseases Unit was founded in Cyprus, with the aim to advance research, and enhance treatment options of inherited cardiovascular diseases in the Cypriot population. One of the missions of the Unit was to document and examine all cases of SDs of young people within the Cypriot population through collaboration with local services. From 2005 to 2015, all cases of SDs in people 1–35 years of age, citizens and permanent residents of the Republic of Cyprus (*n* = 74 SD cases), were recorded and studied.

In this study, all data were collected from Cyprus hospitals where deceased young people were taken, as well as from the Forensic Medicine Service where the post‐mortem examinations of all cases were carried out. According to Cyprus law all SD cases are considered police cases and are investigated by the Police Authorities and the Forensic Medicine Service. Cyprus, as an island with a relatively small and homogeneous population, offers a unique benefit for the detection and recording of all cases of SDs in the Cypriot population.

To achieve comparability with other studies on SD, people over the age of 35 years and infants below the age of 1 year were excluded from the current study. Furthermore, young people who were known drug users or were found at the scene of death with narcotic drugs were excluded from the study. Although it is recognized that these circumstances of death do not preclude the possibility of underlying heart disease complicated with fatal arrhythmia, it was considered preferable to exclude these cases.

Young people who were not permanent residents of the Republic of Cyprus were excluded, and the population analyzed consisted exclusively of Greek Cypriot residents. Therefore, the population analyzed consisted of a relatively homogeneous genetic population, reducing potential confounding due to ethnic diversity and provides a useful basis for future studies investigating familial and genetic contributions to sudden death. There were no SD cases with successful resuscitation.

The study was approved by the Local Scientific Ethics Committee for Medical Research and was conducted in accordance with the Declaration of Helsinki (1989) of the World Medical Association.

### 2.2 Forensic Protocol

As was mentioned before, all cases of SDs in Cyprus are considered police cases according to the law and they are investigated by the police authorities to record the circumstances of death. Subsequently, the cases are reviewed by the Forensic Medicine Service to investigate the cause of SD. The causes of SDs were determined based on the findings of the post‐mortem examination.

In all cases of SDs, the heart, lung, and large vessels of the diseased were preserved in formaldehyde and underwent pathologic anatomical examination for further analysis. A full forensic examination was performed at the time of autopsy, included a macroscopic examination of the body and all organs in order to exclude noncardiac causes of SD, especially cerebral, gastrointestinal, and respiratory causes and acute hemorrhagic or septic shock. To exclude cardiac causes of SD the heart, lung and large vessels of the diseased were examined by an experienced cardiac pathologist following the international guidelines for the autopsy investigation of sudden cardiac death [8, 9]. This examination included the weight of the heart, the pericardium, the dimensions of the heart cavities, the thickness of the heart walls, the heart valves and the origin, course and obstructions of the coronary vessels. Microscopic, histological, and immunobiological examination of the heart was also carried out in all cases. Cardiomyopathic features, fibrosis, or inflammatory infiltration changes as well as the presence of hypertrophy or myocardial disarray were assessed in each case. Depending of the circumstances surrounding the death, toxicologic tests were also performed.

In a significant number of cases, and with the permission of the forensic service, a cardiologist was present at the post‐mortem examination.

### 2.3 Definitions

Sudden Death (SD) was defined as a non‐violent, unexpected death of a seemingly healthy person that occurs within 1 h of the onset of symptoms. If the death was unwitnessed, it was considered as sudden death if the patient was seen in good health 24 h before the incident. Depending on the etiology of SD the cases were described as: (1) Sudden Cardiac Death (SCD), where a cardiac cause of death was found. (2) Noncardiac Sudden Death (NCSD) was defined where a noncardiac cause of SD was found, for example, pulmonary embolism or intracranial hemorrhage, etc. (3) Cases where the post‐mortem and toxicology investigations could not reveal the cause of SD the heart was structurally normal at gross, histological and toxicological examinations and a noncardiac etiology of SD was excluded, were characterized as Sudden Unexplained Death (SUD) or Autopsy‐Negative SUD [4, 5, 6, 7]. The term Sudden Arrhythmic Death Syndrome (SADS) was also used when no cause of death could be established based on autopsy [10, 11, 12].

Based on the official census data in 2011, the population of young Cypriots aged 1–35 years, was estimated at 310 287 people [13].

### 2.4 Statistical Analysis

The statistical analysis was performed using standard spreadsheet platforms (EXCEL) and the statistical package SPSS. For all the variables of the research, a descriptive analysis and an inferential analysis were performed. Exploratory data analysis was performed on all variables, including data distribution and frequency tables as appropriate. For continuous variables, their mean and standard deviation were calculated, while categorical variables are presented as relative percentages and absolute frequencies. Comparisons between groups were made using the *χ* ^2^ test for the categorical variables and *t*‐test and dispersion analysis (ANOVA) for the continuous variables. All assessments were made at the 5% level of significance.

## 3 Results

### 3.1 Sudden Death Population

**Figure 1:**
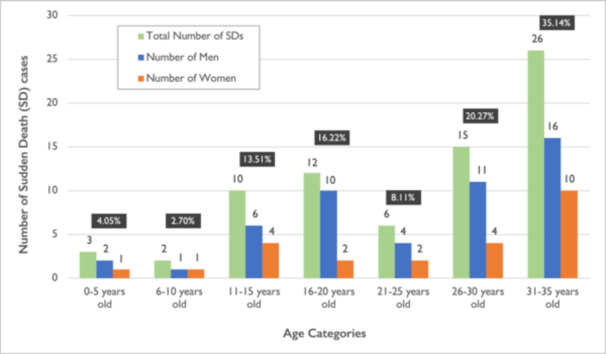
Age and sex distribution of Sudden Death cases in young people 1–35 years old in Cyprus (years: 2005–2015).

Over a period of 11 years, from 2005 to 2015, 74 SD cases of young people aged 1–35 years were recorded in Cyprus. Out of the 74 cases, 50 (67.57%) were males and 24 (32.43%) were females, with a 2.08:1 male‐to‐female ratio. The mean age of sudden death was 24.2 ± 9.1 years, with the mean age for males being 24.9 ± 8.3 years and for females being 22.6 ± 10.5 years. The largest number of SDs was observed in the age group of 31–35 years old (*n* = 26, 35.14%). No significant difference was observed for the age of SD between males and females (*p *= 0.312). The distribution of SD cases by age and sex is shown in Figure 1.

### 3.2 Epidemiological Data

In the present study, the incidence of SD in the young Cypriot population was estimated 2.16 SDs per 100 000 people 1–35 years old, per year.

On average 6.73 ± 0.79 cases of SDs per year were recorded with no significant discrepancies between the years. The most SD cases were recorded during the spring (*n* = 20, 27.03%) and summer months (*n* = 20, 27.03%), and the least during autumn (*n* = 16, 21.62%), while in winter, 18 SD cases (24.32%) were reported. No statistically significant difference was observed in SD cases between the seasons of the year (*p* = 0.90).

Regarding the day of death, most of the SDs were observed on Sundays (*n* = 16, 21.62%) and the least on Thursdays (*n* = 7, 9.45%). No significant difference in SDs cases was observed depending on the day of death (*p* = 0.44).

**Figure 2:**
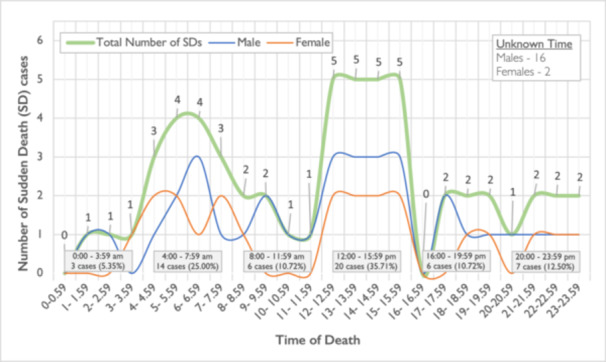
Classification of SDs by hour of the day (circadian variation of SD).

Information on the time of death was available for 56 of the 74 cases of SD. The time the diseased was found dead was used as the time of SD. It was observed that most SDs were recorded between the hours of 12:00–15:59 (*n* = 20, 35.71%) while the least SDs were recorded between the hours 0:00–3:59 (n = 3, 5.35%). In relation to other hours, 25% (*n* = 14) of SD cases were observed during 4:00–7:59 12.50% (*n* = 7) of cases during 20:00–23:59 and 10.71% (*n* = 6) of cases were recorded during both 16:00–19:59 and 8:00–11:59 time periods. There is a statistically significant difference between the time of death, in particular, between the period after noon, morning, and late midnight hours (*p *= 0.001). The classification of SDs by hour of the day is shown in Figure 2.

### 3.3 Etiology of Sudden Death

The cause of death was documented in 53 of the 74 cases of SD reported (71, 62% of SD cases). In 32 cases of SDs (43, 24%) the cause was attributed to cardiac causes and in 21 cases of SDs (28.38%) the cause was attributed to noncardiac reasons. In the remaining 21 cases of SDs (28, 38%), no obvious cause of death was found after post‐mortem, microscopic pathology, histological and immune‐histological examination of the heart and other organs.

According to sex, the majority of SD cases in males were due to cardiac causes (*n* = 25/50, 50% of males), while only 9/50 cases (18%) were due to noncardiac causes. In females, the majority of SD cases were due to noncardiac origin (*n* = 12/24, 50% of females) and only 7/24 (29, 16%) of SDs cases were due to cardiac origin, noting a statistically significant difference between males and females in terms of the cardiac and noncardiac causes of SDs (*p *= 0.017). This information is shown in Table 1.

**Table 1:** Classification of SD cases to cardiac etiology, noncardiac etiology and of unexplained etiology (autopsy negative). Age group analysis.

Sudden death causes	Total number of SDs		Male		Female		SDs cases—age subgroup analysis					
	Number of SDs	%	Number of SDs	%	Number of SDs	%	1–5 Years old	%	6–20 Years old	%	21–35 Years old	%
**Cardiac causes of sudden death**	**32**	**43.24**	**25**	**50.00**	**7**	**29.17**	**1**	**33.33**	**13**	**54.17**	**18**	**38.30**
Hypertrophic cardiomyopathy	11	14.86	11	22.00	0	0.00	0	0.00	6	26.09	5	10.87
Dilated cardiomyopathy	4	5.40	4	8.00	0	0.00	0	0.00	1	4.35	3	6.52
Arrhythmogenic cardiomyopathy	3	4.05	3	6.00	0	0.00	0	0.00	1	4.35	2	4.35
Coronary artery disease‐including recent myocardial infarction	5	6.76	5	10.00	0	0.00	0	0.00	0	0.00	5	10.87
Anomalous origin of coronary arteries	1	1.35	1	2.00	0	0.00	0	0.00	1	4.35	0	0.00
Viral myocarditis	7	9.46	1	2.00	6	25.00	1	20.00	4	17.39	2	4.35
Mitral valve prolapse	1	1.35	0	0.00	1	4.17	0	0.00	0	0.00	1	2.17
**Non**‐**cardiac causes of sudden death**	**21**	**28.38**	**9**	**18.00**	**12**	**50.00**	**2**	**66.67**	**5**	**20.83**	**14**	**29.79**
Hemorrhagic stroke	4	5.41	3	6.00	1	4.17	0	0.00	2	8.70	2	4.35
Pulmonary embolism	8	10.81	1	2.00	7	29.17	0	0.00	1	4.35	7	15.22
Pulmonary aspiration	3	4.05	2	4.00	1	4.17	1	40.00	2	4.35	0	0.00
Aortic aneurysm rupture	3	4.05	3	6.00	0	0.00	1	20.00	0	0.00	2	4.35
Adrenal hemorrhage	3	4.05	0	0.00	3	12.50	0	20.00	0	0.00	3	4.35
**Unexplained cause of sudden death**	**21**	**28.38**	**16**	**32.00**	**5**	**20.83**	**0**	0.00	**6**	**25.00**	**15**	**31.91**
Total	74	100.00	50	100.00	24	100.00	3	100.00	24	100.00	47	100.00

**Figure 3:**
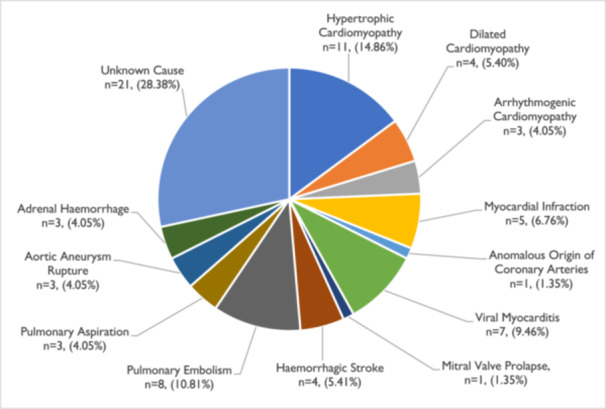
Causes of SD in young people aged 1–35 years in Cyprus.

A detailed representation of the causes of SDs is illustrated in Table 1 and Figure 3. Cardiomyopathies (*n* = 18/74, 24.32%), with predominantly hypertrophic cardiomyopathies, were the leading cause of SD. Pulmonary embolism (*n* = 8, 10.81%), Myocarditis (*n* = 7, 9.46%), and myocardial infarction (*n* = 5, 6.76%) were the second, third, and fourth causes of SDs, respectively. Other causes of SDs were hemorrhagic stroke at 5.41% (*n* = 4), aortic aneurysm rupture, adrenal hemorrhage, and pulmonary aspiration at 4.05% (*n* = 3), respectively. Additional causes of SD were the anomalous origin of coronary arteries and mitral valve prolapse at 1.35%, respectively. The cause of death remained unclear in 21 cases (28.38%).

In men, the main causes of SDs were cardiomyopathies (*n* = 18, 36.00%) and coronary artery disease (*n* = 5, 10.00%). In women, the main causes of SDs were pulmonary embolism (*n* = 7, 29.17%) and myocarditis (*n* = 6, 25.00%). A percentage of 32.00% (*n* = 16) of SDs in males and 20.83% (*n* = 5) of SDs in women remained unclear.

A subgroup analysis of SD cases (Table 1) in very young (1–5 years old), children/adolescents (6–20 years), and adults (21–35 years) revealed distinct patterns. Noncardiac causes of SD were most prominent in the very young group, while cardiac causes predominated in the children/adolescents and adult group. In adults (21–35 years), 38.30% of SD cases were of cardiac origin, 29.79% were noncardiac, and 31.91% remained unexplained.

Cardiomyopathies emerged as the most common underlying cardiac cause in the children/adolescents and adult group. Corornary artery disease was only present in the adult group. Among noncardiac causes, pulmonary embolism was the leading cause of SD in the 21–35 years group.

Within cardiac causes of SDs, cardiomyopathies (*n* = 18, 56,25%) and coronary artery disease (*n* = 5, 15,62%) were the main causes of SDs. Out of noncardiac causes, pulmonary embolism (*n* = 8, 38.10%) and hemorrhagic stroke (*n* = 4, 19.05%) were the main causes of SDs.

### 3.4 Circumstances and Activities Surrounding the Sudden Death

The conditions of SD were studied in 53 of the 74 cases, where there was information about the conditions of SD. The majority of SDs occurred while victims were in their houses (*n* = 33, 62.26%), with 28.30% (*n* = 15) of them performing house activities and 22.64% (*n* = 12) asleep. Fourteen cases (26.42%) of SD cases were related to sports activity, while four cases (7, 55%) occurred during working office hours, and two (3.77%) while driving their car. There is a statistically significant difference between the circumstances of activities at the time of SD, in particular, those that occurred in the house against those during working office hours and during driving a car *(p *= 0.0001). This information is shown in Table 2.

**Table 2:** Place of death and activity at the time of sudden death (Based on 53 cases of SDs for which information was available).

Place of sudden deaths (SD) and activity	Number of SDs	%
**At home**	**33**	**62.26**
House activities	15	28.30
Sleep	12	22.64
Eating	4	7.55
Shower	2	3.77
**Sports activities**	**14**	**26.42**
Football match	5	9.43
Other sports activities	9	16.98
**Working office hours**	**4**	**7.55**
**Driving**	**2**	**3.77**
Total	53	100.00

## 4 Discussion

The sudden and unexpected loss of a young person is a tragic event for both the family and the community. It raises several questions about the cause of sudden death in a previously asymptomatic individual and the possibility of an underlying condition affecting other family members. Consequently, such cases are usually investigated through a detailed post‐mortem examination to determine the exact cause of death.

Several studies in the developed world including Europe, USA and Australia [1, 2, 3, 4, 5, 6, 7, 14, 15, 16, 17], have attempted to investigate the epidemiology of sudden deaths and the published data have identified various causes with a variety of prevalences across different studies. These discrepancies can be attributed to the methodology of each study but also to national, geographical and possibly genetic differences of the populations studied. In most reports, the study was based on a retrospective analysis of death certificates and autopsy data.

The uniqueness of the present study is that: (1) It's a prospective study recording and studying all sudden deaths of young victims age 1–35 years old over a period of 11 years; (2) all cases of SD, and not just SCD cases were included and studied; and (3) is a nationwide study, organized on a specific national and geographical area such as the Republic of Cyprus, an island located at the south end of Europe and the east end of Mediterranean Sea with a good socioeconomical level similar to other European countries. The analysis of the data for this study began immediately with becoming aware of the existence of a case of sudden death in a young person. Further data were obtained by post‐mortem examination, then by family and past medical history and when possible, from a clinical examination of close family relatives. Additional information was collected by discussion and analysis of histopathological findings with pathologists.

In this study, the incidence of SD in the young Cypriot population was 2.16 SDs per 100 000 people 1–35 years old, per year. This percentage is consistent with that of other studies. Specifically, a study investigating SDs in the Netherlands population [4] from 1996 to 2006 showed that the incidence of SDs in people < 40 years of age was 2.07 per 100 000 per year. A similar study in Denmark [6, 14] showed 2.8 SDs per 100 000 young people per year, and a study of sudden cardiac deaths in Ireland [18] showed an incidence of 2.85 cases per 100 000 young people per year. In a South European population study [19] during a 5‐year period, 159 SD were identified corresponding to an annual incidence of 2,4 SD per 100 000 people years. A study of SDs in Greece from 2002 to 2010 showed that the incidence of SDs was 2.7 per 100 000 young people per year [12]. In the region of Epirus in Greece [20], the estimated annual rate of SCD in the population 1–35 years old was 1.78 per 100 000 people years.

According to sex, likewise in other SD studies [9, 10, 21], the incidence of SD in young adults was higher in males compared to females (Male: 67.60%, Female: 32.40%) with a ratio of 2.08–1. This fact has been confirmed by previous studies. In a retrospective autopsy study of SDs in young south European population [19] 72, 3% of SD victims were males. In the eastern part of Sydney, Australia [16, 17], 70.7% of SD in young people were male. In a study of SCD in Czech Republic [15], the incidence of SCD in men exceeded the incidence in women four times, whereas in Denmark and Australia, the male incidence of SCD was only double that of females [6, 16, 17, 22]. The atheroprotective effect of estrogens has been well established [23], however other protective mechanism must be involved as men seem to be more vulnerable to coronary artery disease.

Although the highest number of SDs was observed in the age group of 31–35 years, no statistical difference was observed for the age of SDs between males and females.

In this study, the highest number of SDs was observed during the summer and spring months (*n* = 20, 27.3% respectively), and the lowest number of SDs was observed in the autumn (*n* = 16, 21.62%) and winter months (*n *= 18, 24.32%). Exposure to high temperatures (> 41°C) during summer in Cyprus is considered to be a possible factor influencing morbidity and mortality from cardiovascular diseases, while winter in Cyprus is mild. More SDs were observed on Sundays (*n* = 16, 21.62%) and fewer on Thursdays (*n* = 7, 9.45%). This difference in terms of seasonal and weekly distribution of SDs was not statistically significant (*p* = 0.87 and *p* = 0.44, respectively). These findings are in line with an earlier study of SDs in the Greek population [5]. Conclusions regarding the seasonal distribution of SDs differ, where other studies report a reduction in the incidence of SDs in the summer months [24, 25], while others report a slightly higher number of sudden cardiac deaths (SCDs) in the summer months, however, these studies had different age groups of SD between 20 and 75 years old.

A circadian fluctuation was observed at the time of SD. Most deaths were recorded in the afternoon 12:00–15:59 (*n* = 20, 35.71%) and in the morning hours 4:00–7.59 (*n* = 14, 25.00%). Fewer deaths were recorded in the evening (*n* = 7, 12.5%) and after midnight hours (*n* = 3, 5.35%). This difference in the hours that SD occurred was statistically significant (*p* = 0.001). Circadian variation of SDs was also observed in other SD studies, which reported higher incidence of SDs episodes between 8:00–12:00 [25, 26]. This circadian variation is known in heart disease and especially in acute coronary syndromes. A study of acute coronary events in the Cypriot population showed an increased incidence of episodes in the prenoon hours 6:00 am–12:00 p.m [27]. Possible interpretations are the sudden increase in heart rate, physical activity, and activity of the autonomic nervous system when waking up in the morning [26, 27, 28, 29].

The present study showed that SDs in young people are not always due to cardiac causes, and in a significant number of cases, it is not possible to determine the cause of SD. In the young Cypriot population, 43.24% (*n* = 32/74) of SD cases were due to cardiac causes, 28.38% (*n* = 21/74) were attributed to noncardiac causes, and in 28.38% (*n* = 21/74), no obvious cause of death was identified even after post‐mortem, microscopic pathological, histological, and immunohistological examination of the heart and other organs. Data concerning the etiology of SD vary in international literature [2, 3, 4, 5, 9, 16, 17, 30]. In a 10‐year study of SDs, in neighboring Israel, individuals under 40 years of age, underlying cardiac disease were found in 73.00% of cases and noncardiac disease in 18.00% [2]. In Australia, over a 10‐year period, cardiac causes accounted for 56.40% of SDs and noncardiac causes for 39.30% which differs from ours [16, 17]. A study from Greece showed that 65% of SDs in the young were due to cardiovascular causes, 17.00% to noncardiac causes, and 18.00% to unexplained causes [12]. Variations in the etiology of SD in the young are possibly due to geographic, ethnic, socioeconomic, and cultural patterns influencing disease and death in different populations, as well as differences in study design and the age ranges included.

A considerable proportion of SD cases in the young remains of unknown cause even after post‐mortem examination (4.30%–8.00%). Apart from channelopathies, subclinical forms of inherited structural cardiovascular diseases are suspected in these cases. According to Anastasakis et al., clinically guided genetic screening has a significant diagnostic yield and may identify cases where arrhythmia syndromes have been missed [12].

Among the cardiac causes of SDs, cardiomyopathies were the leading cause, accounting for 56.25% of SD cases in Cyprus, followed by myocarditis (21.85%) and coronary artery disease (myocardial infarction) in 15.62% of SCD cases (Table 1, Figure 3). A possible explanation for this is the phenomenon of closed societies. As an island with a relatively closed population, Cyprus may have a higher incidence of hereditary diseases compared to countries with more open, diverse populations.

The relatively low proportion of CAD‐related deaths in Cyprus contrasts with findings from Western and Northern Europe and Australia, where atherosclerotic coronary disease is the dominant cause of cardiac SD, accounting for 33.00%–38.00% of cases [6, 7, 11, 14, 15, 19, 22]. For example, CAD accounted for 24.00% of SDs in Australia [22] and 38.00% of SDs among individuals aged 2–40 years in the Czech Republic [15]. These findings may reflect differences in cardiovascular risk profiles and adherence with healthy lifestyle recommendations for the reduction of atherosclerosis. The Czech Republic, for instance, is recognized as one of the highest‐risk regions for CAD in Europe. In contrast, Cyprus, as a Mediterranean country, has a relatively lower incidence of coronary artery disease [29], accounting for only 15.62% of SDs in the young population. The dietary patterns and physical activity levels traditionally associated with Mediterranean populations may contribute to this finding. Furthermore, as mentioned previously, the relatively closed genetic pool of the Cypriot population may lead to a higher prevalence of inherited cardiac diseases, such as cardiomyopathies, further reducing the relative contribution of CAD. These findings highlight the geographical, environmental, and socioeconomic factors influencing the characteristics and etiology of SDs across different countries.

Among the noncardiac causes of SD, pulmonary embolism and hemorrhagic stroke were the leading contributors, accounting for 38.10% (*n* = 8) and 19.05% (*n* = 4) of cases, respectively. In contrast, epilepsy was the most common noncardiac cause of SD in other studies [4, 31]. In a South European population [19], respiratory infections were the leading cause of noncardiac SD (55.30%), followed by hemorrhagic stroke (8.50%), generalized tonic‐clonic seizures (8.50%), and subarachnoid hemorrhage (4.20%). Ethnic differences may also contribute to variations in the causes of SD between countries.

In males, the main cause of SD was due to cardiac causes (*n* = 25/50, 50%) while only 18.00% (*n* = 9/50) of males SD were due to noncardiac causes. In women, the main cause of SD was noncardiac causes (*n* = 12/24, 50%), and only 29.17% (*n* = 7/24) of women were due to cardiac causes of SD. The difference in the causes of SD between men and women was statistically significant (*p* = 0.017). Cardiomyopathies and coronary artery disease as a cause of SD were found only in men, while pulmonary embolism and myocarditis as a cause of SD were observed mostly in women.

In 28.38% (*n* = 21/74) of SD cases, the cause of SD remained unclear. More specifically, 32% of SDs in men (*n* = 16/50) and 16.67% of SDs in women (*n* = 5/24) remained unclear. In published studies, the percentage of unexplained etiology of SDs varies from 6% to 35% [1, 2, 3, 4, 5, 6, 15, 21, 22]. Our findings are consistent with the respective data from Denmark [6, 31] and Greece [12]. It is known that SD can occur in the absence of structural changes in the heart, as in familial electrical syndromes. A relatively significant portion of SDs remain unexplained after post‐mortem analysis, referred to as sudden arrhythmic death syndrome (SADS). Population‐based studies [12] suggest a detection rate for four‐gene molecular autopsy up to 15%–20%. Relative studies [10, 11, 12, 32, 33, 34, 35, 36] managed to detect an inherited cardiac disease in 22%–53% of the families of SADS victims. Consensus documents [33, 34, 37, 38], recommend retaining samples and perform postmortem genetic testing in sudden unexpected death in the young with a normal autopsy when an inherited cardiac condition is suspected. Genetic testing will help to clarify such cases. Unfortunately, genetic research was not available at the time of the study due to its high cost. A survey of the European Heart Rhythm Association on 24 European countries showed on average that, autopsy was performed in 43% of SUD and post‐mortem genetic testing was requested on average in 37% of SADS [32].

Despite the small number of cases of SDs, an attempt was made to study the conditions at the time of SD. In 62.26% (*n *= 33) of the cases, the death occurred while the diseased patients were in their houses (Table 2), with the majority of those dying during the execution of household chores (*n *= 15, 28.30%) and during sleep (*n* = 12, 22.64%). In a percentage of 26.42% (*n *= 14), SD occurred during sports activities (Table 2). In the study by Margey et al. [18] in Ireland, 45% of SDs occurred during non‐strenuous activity or sleep, and only 7.70% during exercise. In the study of Wisten et al. [7] in Sweden, SDs occurred at a rate of 40% during sleep and only 14% during daily activities. In a recent paper in SD cases in young people in the South European population [19], 55.4% of SD happened while people were at home, 37.5% at rest, 17.5% during sleep, and only 15% after exertion. The proportion of cases dying during sports activity in this study (26%) appears to be high compared to other studies where the majority of deaths occurred during rest, sleep, or light activity [4, 5]. For example, in Denmark and Australia, only 11% and 15% of young people, respectively, died during sports activities [4, 5, 16, 17, 22]. Although the number of cases is small to exclude significant conclusions, a possible explanation is environmental. Cyprus experiences prolonged periods of high temperatures and humidity, which may increase cardiovascular strain during physical exertion. Supporting this hypothesis is the observation that the highest number of SDs occurred during the summer months. Furthermore, the high rate of participation in organized sports, particularly football, among young people in Cyprus may have contributed to the greater proportion of activity‐related SDs observed.

### 4.1 Strengths and Weakness of the Study

This study has several notable strengths. It represents the first nationwide, prospective analysis of SDs in young people in Cyprus, covering all cases over a 11‐year period. Few studies globally have investigated the etiology and frequency of SD in young populations at a national level, encompassing the total population of a country. Cyprus's geographical characteristics and small population facilitated the identification and recording of all SD cases within this defined population. The mandatory reporting and investigation of all SDs in Cyprus, along with comprehensive post‐mortem examinations, including histological and toxicological analyses, ensured data completeness and minimized case under‐ascertainment. Studying a relatively homogeneous population of Greek Cypriot permanent residents reduced confounding from ethnic diversity and provided valuable insights into population‐specific and potentially hereditary patterns of SD. The findings, particularly the predominance of cardiomyopathies over coronary artery disease as the leading cause of SD, enable meaningful comparisons with international studies and highlight the possible influence of genetic, environmental, and lifestyle factors on SD etiology.

The limitations of this study include the small number of SD cases, which resulted due to the small population size of Cyprus and the corresponding young Cypriot population aged 1–35 years (310 287 individuals). Moreover, as the study population consisted exclusively of Greek Cypriot permanent residents, the findings may not be fully generalizable to more ethnically diverse populations. Another limitation is the exclusion of SD cases in known drug users; detailed analysis of these cases identify underlying heart disease, which may have led to a fatal arrhythmia. A major limitation of this study is the absence of genetic testing of SD victims, primarily due to the high cost of the genetic examination during the study period. While many countries still lack the capacity for routine genetic testing in all SD cases, advances in next‐generation sequencing technologies now allow rapid and cost‐effective screening of large gene panels, including cardiomyopathy genes [31, 36]. However, whole‐exome sequencing presents challenges in the identification of a large number of genetic variants of unknown significance that need to be interpreted with caution, given the dramatic clinical consequences of potential false‐positive results. Recent expert consensus statements [33, 34] recommend the consideration of targeted ion channel genetic testing in SADS cases and advocate comprehensive screening when circumstantial evidence suggests diagnoses such as LQTS or CPVT specifically. Clinically guided genetic screening could clarify underlying inherited cardiac diseases, particularly arrhythmia syndromes, in approximately 20%–25% of autopsy‐negative and unexplained etiology SD cases.

## 5 Conclusions

In this study, it was possible to record and study, in a period of over 11 years, all SDs of people 1–35 years old, at a national level, using the Cypriot SD registry. The incidence of SD in the population we studied was 2.16 cases of SD per 100 000 young people per year. We have demonstrated that SDs in young people are mainly due to cardiac causes (cardiomyopathy, myocarditis and coronary artery disease) but not exclusively, as there were many SDs from noncardiac causes.

Despite the absence of genetic testing of sudden death victims, the uniqueness of this study to record and study all SDs immediately after the event, at a national level and in a specific geographical area such as the island of Cyprus, we believe it provides reliable and useful information on the prevalence and etiology of SDs in young people in a country located in the east Mediterranean area at the south‐east of Europe. Future studies incorporating molecular autopsy and family screening could further elucidate the role of inherited cardiac conditions in unexplained cases.

## Data Availability

The data that support the findings of this study are available on request from the corresponding author. The data are not publicly available due to privacy or ethical restrictions.
